# Liangyi Gao extends lifespan and exerts an antiaging effect in *Caenorhabditis elegans* by modulating DAF-16/FOXO

**DOI:** 10.1007/s10522-019-09820-7

**Published:** 2019-07-22

**Authors:** Liling Zeng, Chen Sun, Zhong Pei, Tianchan Yun, Shaoyi Fan, Simei Long, Tengteng Wu, Ziwen Chen, Zhimin Yang, Fuping Xu

**Affiliations:** 1 The Second Clinical College of Guangzhou University of Chinese Medicine, Guangzhou, China; 2 Guangdong Provincial Hospital of Chinese Medicine, 111 Da De Rd, Yuexiu District, Guangzhou, 510120 Guangdong Province China; 3 Department of Neurology, National Key Clinical, Department and Key Discipline of Neurology, the First Affiliated Hospital, Sun Yat-sen University, Guangzhou, China

**Keywords:** Antiaging, Liangyi Gao, Traditional Chinese Medicine, *Caenorhabditis elegans*, DAF-16/FOXO regulation

## Abstract

Liangyi Gao (LYG), a traditional Chinese medicine, is composed of *Ginseng* and *Radix Rehmanniae Preparata,* both of which have been shown to have antiaging properties. In Eastern countries, LYG is used to delay functional declines related to aging and has an obvious antiaging effect in clinical practice. However, little data from evidence-based medicine is available regarding whether LYG is beneficial overall, particularly with respect to lifespan, and how LYG functions. To address these issues, *Caenorhabditis elegans*, a useful organism for such studies, was employed to explore the antiaging effect and mechanism of LYG in this study. The results showed that LYG could obviously extend lifespan and slow aging-related declines in N2 wild-type *C. elegans*. To further characterize these antiaging effects and stress resistance, reproductive tests and other aging-related tests were performed. We found that LYG enhanced resistance against oxidative and thermal stress, reproduction, pharynx pumping, motility and growth in N2 wild-type *C. elegans*. In addition, we analyzed the mechanism for these effects by measuring the activity of superoxide dismutase (SOD) and the expression levels of aging-related genes. We found that LYG enhanced the activities of antioxidant enzymes and upregulated the genes daf-16, sod-3 and sir-2.1, which mediated stress resistance and longevity. In conclusion, LYG had robust and reproducible life-prolonging and antiaging benefits in *C. elegans* via DAF-16/FOXO regulation.

## Introduction

Aging is a complex process characterized by the progressive failure of maintenance and repair pathways. Aging leads to dysfunction, disease, and ultimately, death, which has long been a concern (Blagosklonny and Hall 2009). Although people have attempted to explore the mechanisms of aging, these mechanisms are complicated, and a specific antiaging intervention is still not known (Chung et al. 2011; Mikhelson and Gamaley 2012; Chen et al. 2013; Isobe 2016).

As a complementary medicine, traditional Chinese medicine (TCM) has 5000 years of history and has attracted much attention for its medical efficacy as well as its preventative functions (Zhou 1982; Chen 2015; Liu et al. 2017). Recently, numerous studies have indicated that many TCMs have an array of antiaging effects (Liang and Yin 2010; Wan et al. 2014; Hsu et al. 2015). Liangyi Gao (LYG) is a TCM formula described in *Jing Yue’s Complete Work*, which is mainly composed of 2 ingredients, *Panax Ginseng* and *Radix Rehmanniae Preparata. Ginseng* combined with *Rehmannia glutinosa* is a classic Chinese herbal prescription that has invigorates Qi and nourishes blood; this prescription is now commonly used to treat weakness or chronic debilitating diseases. Both *Ginseng* and *Rehmannia glutinosa* have been demonstrated to have antiaging effects in previous studies (Yang et al. 2017; Bai et al. 2018). In addition, ginsenoside Rg1, an active ingredient in *Ginseng*, has effects that include antiaging as well as improving immunity (Cheng et al. 2005; Zhou et al. 2011; Chen et al. 2014; Tang et al. 2015; Zhou et al. 2015); additionally, catalpol, an active ingredient in *Rehmannia glutinosa*, also exhibits antiaging effects (Zhang et al. 2013; Seo et al. 2015). Moreover, we have drawn from more than 10 years of clinical experience, which has shown that LYG has strong antiaging effects. Since little data from evidence-based medicine is available about whether LYG is beneficial overall, we performed this study to confirm its benefits and to explore the mechanism of its antiaging effects using *C. elegans*, which has become a widely accepted model for aging research because of its short lifespan, morphology, ease of culture, and ease of genetic manipulation (Guarente and Kenyon 2000; Wang et al. 2014). Moreover, since *C. elegans* shares nearly two-thirds of its genes with humans, it is used as a well-characterized experimental system for aging and aging-associated disease research (Sluder and Baumeister 2004).

To investigate whether LYG has an antiaging effect, we performed lifespan assays and stress resistance assays, and we assessed other aging-related properties with wild-type *C. elegans* N2 (Bristol). Furthermore, to examine the underlying mechanisms, we analyzed the effects of LYG on the activity of superoxide dismutase (SOD) and on the expression levels of aging-related genes.

## Methods

### Drug preparation

The LYG formula contains a 1:2 ratio of *Ginseng* and *Radix Rehmanniae Preparata*. The formula was extracted by water, and its concentration was determined by the amount of the crude drug in solution. The *Ginseng* and *Radix Rehmanniae Preparata* were provided by *Kangmei Pharmaceutical* (Guangzhou, China), and the LYG was produced according to the detailed specifications of *Jing Yue’s Complete Work*. Ginsenoside Rg1 (Lot: A0503AS) and catalpol (Lot: 50728AS) were provided by *Meilunbio* (Dalian, China). All drugs were dissolved in dimethylsulfoxide (DMSO) and were diluted in *E. coli* OP50 solution when the drugs were added during the preparation of agar plates. Detailed information about the drugs used in the different groups is shown in Table 1.

**Table 1:** The components, dose proportions, and original concentrations of the drugs in the different groups

Groups and drugs	Components	Dose proportion	Original concentration (mg/ml)^a^
LYG	*Ginseng* and *Radix Rehmanniae Preparata*	1:2	1 mg/ml
Rg1	Ginsenoside Rg1	–	150 µg/ml
Catalpol	Catalpol	–	150 µg/ml
Rg1 + Catalpol	Ginsenoside Rg and catalpol	1:1	150 µg/ml
Control	DMSO	–	Equal to the other groups

^a^The concentrations of the drugs used to treat N2 wild-type nematodes in this study were determined by a dose–effect curve and were chosen based on the dose with the greatest effect on the lifespan of N2 wild-type nematodes; DMSO, dimethylsulfoxide

### *Caenorhabditis elegans*: strains and maintenance

The Genetics Center (CGC) at the University of Minnesota (Minneapolis, MN, USA) provided wild-type *C. elegans* N2 (Bristol), the CF1553 *C. elegans* strain, TJ356 *C. elegans* strain, and *E. coli* OP50. *C. elegans* strains were maintained at 20 °C on solid nematode growth medium (NGM) plates seeded with *E. coli* OP50.

### Lifespan analysis

Age-synchronized N2 nematodes were transferred to NGM plates containing drugs or vehicle control (DMSO). Two NGM plates containing 25 worms per plate were established for each group, and the worms were transferred to a new NGM plate every day during the first 7 days to prevent new eggs from causing a disturbance. The survival rate was assessed every other day until death. The nematodes were considered dead when they failed to respond to touch using a platinum loop (touch-provoked method). The lifespan assay was repeated in more than two independent trials.

### Assessment of stress resistance

Age-synchronized N2 worms were bred on NGM plates with drugs or vehicle control (DMSO). For the heat tolerance assay, the adult day 4 worms (n = 50) were transferred to fresh plates and then incubated at 37 °C. Survival was recorded every hour until all worms died. Oxidative stress tolerance was assessed as described previously with minor modifications (Munoz and Riddle 2003). Briefly, the adult day 4 worms (n = 50) were placed on plates containing various concentrations of hydrogen peroxide (from 0 mM to 1 mM, in intervals of 0.2 mM), and then survival was recorded over 15 h. The survival of worms was determined by touch-provoked movement, as described above. Each test was performed at least two times.

### Measurement of SOD activity

To assess the antioxidant enzyme activity, worm homogenates were prepared. Briefly, wild-type worms (n = 50) were collected from plates with M9 buffer on adult day 5 and were washed 3 times. Then, the collected worms were resuspended in homogenization buffer (10 mM Tris–HCl, 150 mM NaCl, and 0.1 mM EDTA, pH 7.5) and homogenized with an ultrasonic wave method on ice. SOD activity was spectrophotometrically measured by analyzing the decolorization of formazan that results from an enzymatic reaction between xanthine and xanthine oxidase. A Total Superoxide Dismutase (T-SOD) Assay Kit (Hydroxylamine method) and a Total Protein Assay Kit (with standards: BCA method) were used for the determination of the protein concentration and SOD activity, respectively, and were purchased from *Nanjing Jiancheng Bioengineering Institute* (Nianjing, China). The procedure was performed in strict accordance with the manufacturer’s protocol.

### Measurement of aging-related factors

Age-synchronized N2 worms were bred on NGM agar plates with or without drugs. On the fourth day of adulthood, single worms were transferred to fresh plates, and their pharynx contractions were counted under an inverted microscope for 10 s (n = 10). For the reproduction assay, 5 worms were raised from embryos. L4 larvae were individually transferred to a fresh plate every day to distinguish the parent from the progeny. The progeny was counted at the L2 or L3 stage. For the growth alteration assay, photographs were taken of adult day 4 worms, and the body length of each animal was analyzed by Nikon software (*Nikon*, Japan). For the body movement assay, on the seventh day of adulthood, single worms were transferred to fresh plates, and their body movements were recorded under an inverted microscope for 20 s. The body movements of the animals were analyzed by Nikon image software, and the data are expressed as the total distance travelled. All the tests were repeated more than two times. Lipofuscin, an endogenous marker of cellular damage during aging (Brunk and Terman 2002), was also assessed on the 10th day of adulthood, and the autofluorescence level of lipofuscin was observed under a fluorescence microscope. The fluorescence intensity was quantified using ImageJ software.

### Fluorescence microscopy and visualization

Age-synchronized transgenic nematodes, including the CF1553 strain that contained a SOD-3::GFP reporter and the TJ356 strain that contained a daf-16::GFP reporter, were maintained in the presence or absence of LYG. On the third day of adulthood, the nematodes were exposed to heat shock at 37 °C for 2 h and allowed to recover at 20 °C for 4 h. Prior to microscopic observation, transgenic animals were anesthetized with sodium azide (2%) and mounted on a 2% agarose pad. The GFP fluorescence of GFP-expressing populations was directly observed under a fluorescence microscope (*Nikon*, Japan). To determine the protein expression levels, photographs of the transgenic worms were taken and analyzed using ImageJ software. All experiments were performed in triplicate.

### Quantitative analysis of aging-related genes in *C. elegans*

Synchronized *C. elegans* were treated with or without LYG for 3 days at 20 °C. The worms were collected, washed three times with M9 buffer, transferred to 1.5 ml RNase-free microfuge tubes, and pelleted by centrifugation at 3000 rpm for 1 min. For every sample containing approximately 600 worms, 1 ml TRIzol reagent was added (*TaKaRa*, Beijing, China); the samples were vortexed and then fully homogenized. For total RNA extraction, 200 ml of chloroform was added, and the worm suspension was vigorously shaken and centrifuged at 12,000×*g* for 15 min. The total nematode RNA in the supernatant was isolated using isopropanol and washed with 75% ethanol. The RNA concentration was quantified using a Nanodrop spectrophotometer. Complementary DNA was produced using random 6-mer and oligo (dT) primers (*TaKaRa*, Beijing, China) according to the manufacturer’s protocol. Quantitative real-time polymerase chain reaction (qPCR) was performed, and SYBR green (*TaKaRa*, Beijing, China) was used as the detection method. Act-1 was used as a reference gene, and the expression levels of each mRNA relative to the act-1 gene were calculated with the comparative 2^−ΔΔCT^ method. The experiment was repeated in triplicate.

### Statistical analyses

GraphPad Prism 6.0 was used for statistical analyses. For the lifespan assay, Kaplan–Meier survival analysis was conducted, and *P* values were calculated using the log-rank test. A Student’s *t* test was performed to compare two datasets. All results are expressed as the mean ± the standard error of the mean (SEM). Values of *P *< 0.05 were considered significant.

## Results

### Effect on lifespan extension and stress resistance

**Fig. 1:**
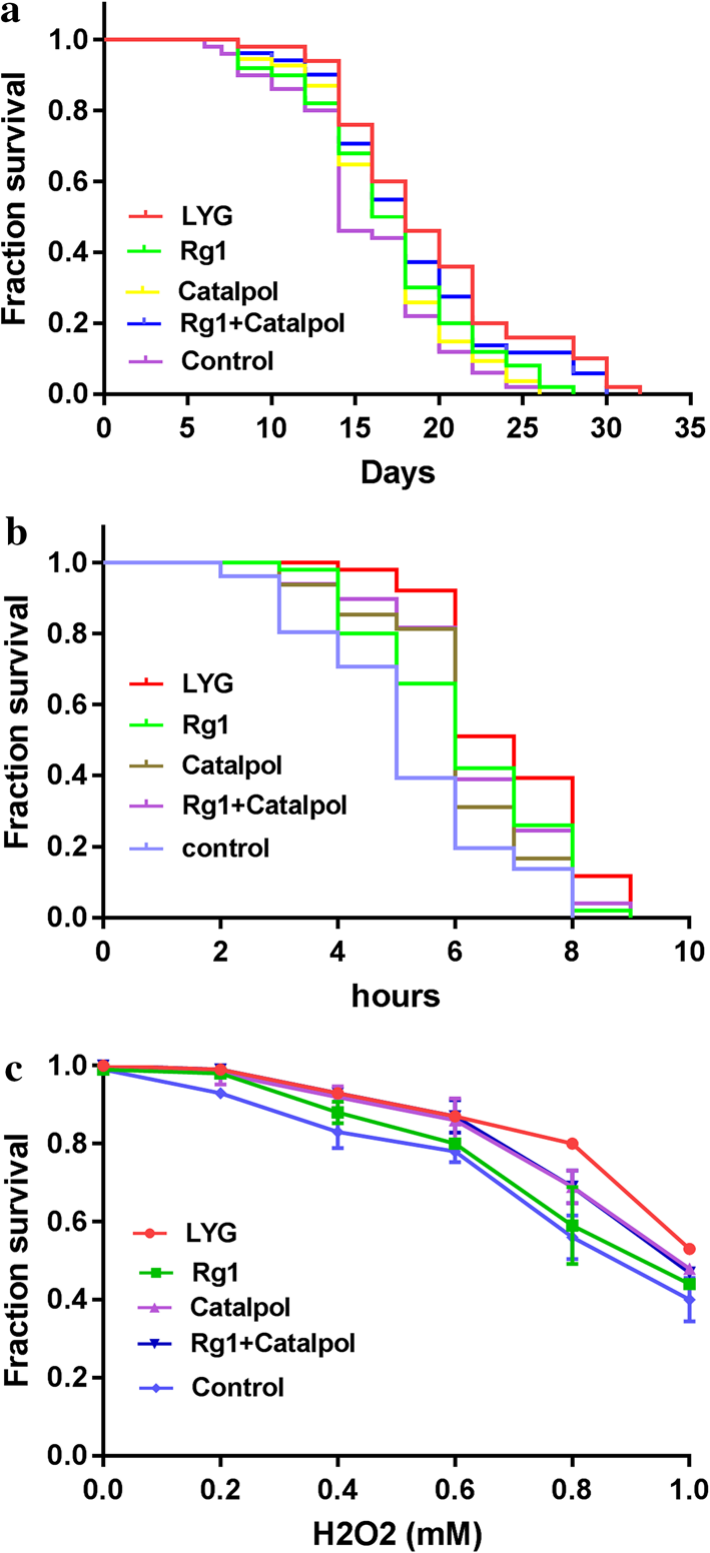
Effect on the lifespan and stress resistance in *C. elegans* N2. **a** Effect on lifespan extension. **b** Effect on heat stress resistance. **c** Effect on oxidative stress resistance

To evaluate the lifespan-extending properties of LYG, lifespan assays were performed using wild-type worms. Herein, we found that worms exposed to LYG showed a higher rate of increase in lifespan than the worms exposed to Rg1, catalpol, or the control; additionally, the Rg1 and catalpol combination treatment showed a benefit similar to that of LYG treatment (shown in Fig. 1a and Table 2). To evaluate stress resistance, we performed heat stress assays and oxidative stress assays. As shown in Fig. 1b, compared to the worms in the control group, worms fed 1 mg/ml LYG had a significantly increased mean lifespan during heat stress (*P *< 0.01). Resistance to oxidative stress was examined by exposing animals to hydrogen peroxide, an intracellular free radical-generating compound. The results also showed that LYG-treated wild-type worms lived longer than the control worms during hydrogen-peroxide-induced oxidative stress (Fig. 1c, *P *< 0.05).

**Table 2:** Effect on lifespan extension

	Mean lifespan^a^	Maximum lifespan	Decline in mean lifespan (%)^b^	*P* value^c^
LYG	19.44 ± 0.80	32	–	–
Rg1	17.08 ± 0.69	28	12.1	0.03*
Catalpol	16.85 ± 0.57	26	2.3	0.00***
Rg1 + Catalpol	18.27 ± 0.75	30	6.0	0.29
Control	15.7 ± 0.64	26	19.2	0.00***

^a^Mean lifespan is presented as the mean ± SEM

^b^Decline in mean lifespan compared with the LYG group (%)

^c^Statistical significance of the difference between the survival curves was determined by the log-rank test using Kaplan–Meier survival analysis. Differences compared to the LYG group were considered significant at *P < 0.05 and ***P < 0.01

### Effect on aging-related factors

**Fig. 2:**
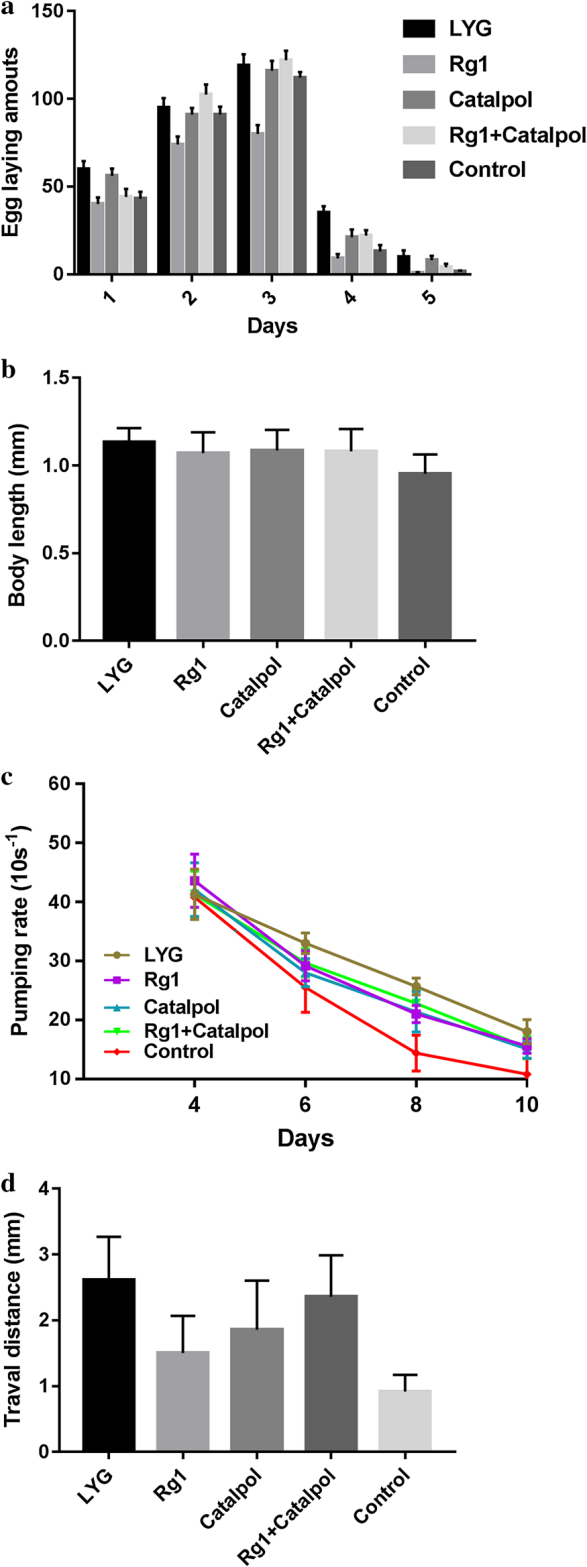
Effects on aging-related factors. **a** Effect on reproduction. **b** Effect on body length. **c** Effect on pharyngeal pumping. **d** Effect on body movement

**Fig. 3:**
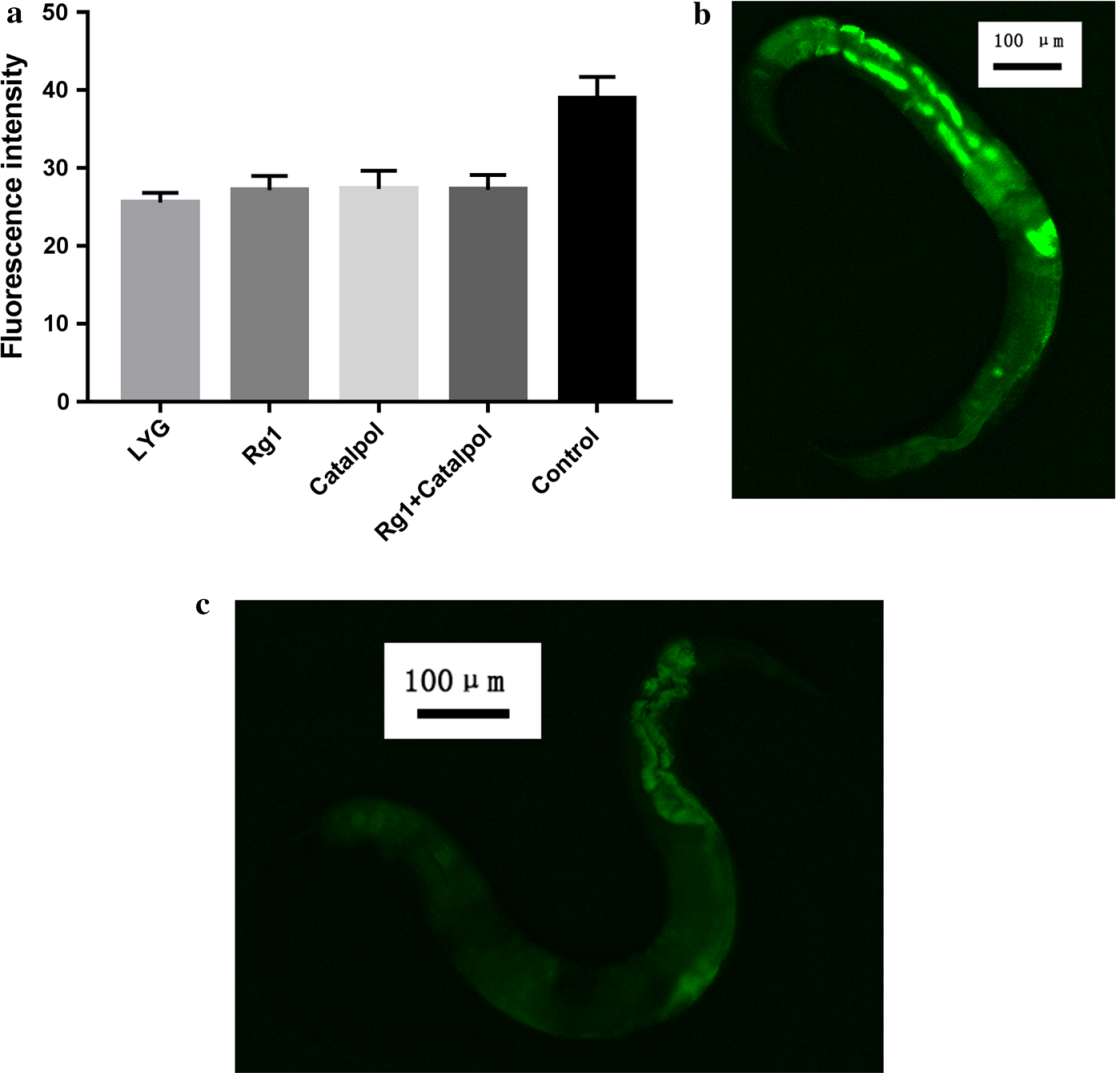
Effect on lipofuscin accumulation. **a** The lipofuscin accumulation was lowest in LYG-treated worms, and the difference compared to the control-treated worms was significant (*P *< 0.001). **b** Fluorescence intensity of intestinal lipofuscin in the control worms. **c** Fluorescence intensity of intestinal lipofuscin in the LYG-fed worms

Previous studies have suggested that longevity is closely connected with reproduction, pharynx pumping, body size and motility in many species, including in *C. elegans* (Zhang et al. 2013). In this study, we showed that LYG treatment, as well as the Rg1 and catalpol combination treatment, significantly increased the total progeny number compared to that in the control group (shown in Fig. 2a, *P *< 0.05). It was especially interesting that the LYG treatment delayed the decline in reproduction and significantly increased the progeny number during the later stage of reproduction (shown in Fig. 2a, during days 4 and 5), suggesting that LYG treatment could delay reproductive decline. In addition, a small but significant change in the body length of worms was detected after LYG exposure (shown in Fig. 2b, *P *< 0.05), suggesting that the antiaging effect of LYG is dependent on growth as well as on fertility. Then, we measured the rate of pharyngeal pumping to estimate the muscle activity and the motor ability of the worms. As shown in Fig. 2c, the rate of pharyngeal contractions gradually declined with increasing age, and this age-associated decline was delayed by LYG treatment. Furthermore, we evaluated whether LYG affected age-associated changes in *C. elegans*, such as body movements. To estimate the health span of worms, we recorded the distance traveled in 20 s. As shown in Fig. 2d, compared to the control group, the LYG group exhibited a significant increase in body movement (*P *< 0.01), suggesting that the functional aging of worms is strongly delayed by LYG. We measured the autofluorescence level of lipofuscin, and the results revealed that compared to control worms, the LYG-treated worms exhibited significantly attenuated fluorescence intensity from intestinal lipofuscin (34.20% decrease; *P *< 0.001, Fig. 3).

### Mechanism of LYG treatment effects on the lifespan of *C. elegans*

#### Effect of LYG on antioxidant enzyme activity

**Fig. 4:**
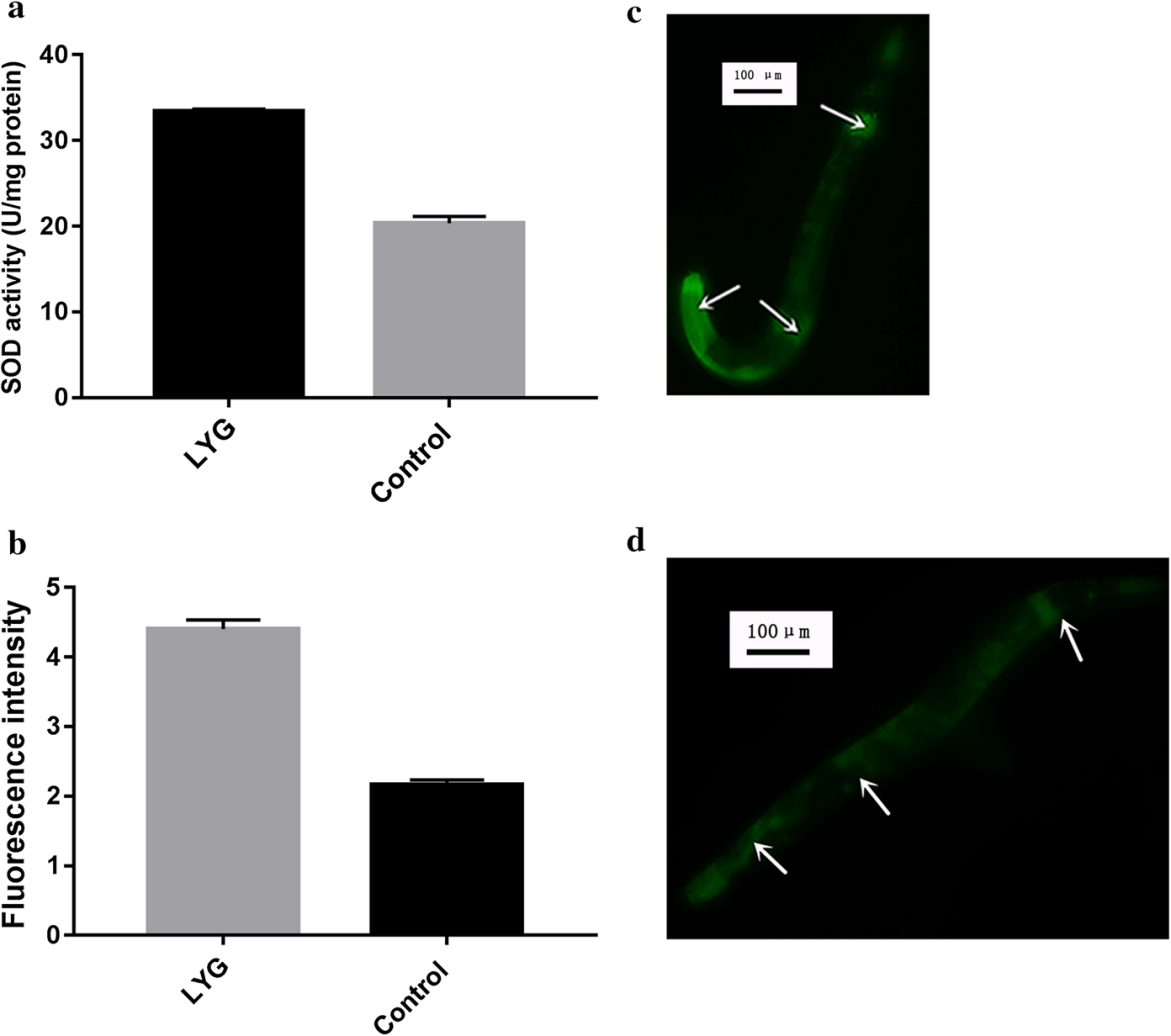
**a** Effect of LYG on antioxidant enzyme (SOD) activity. **b** The fluorescence intensity of SOD-3::GFP expression in LYG-treated CF1553 worms was significantly stronger than that in the worms in the control group. **c** Fluorescence intensity of SOD-3::GFP expression in LYG-treated CF1553 worms. **d** Fluorescence intensity of SOD-3::GFP expression in control CF1553 worms

To verify the possible mechanism of LYG-mediated lifespan extension and stress tolerance elevation, the activities of stress resistance proteins were investigated in wild-type worms. In the present study, we measured the activities of antioxidant enzymes such as SOD using prepared worm homogenates. As noted in Fig. 4a, compared to that in the control group, SOD was significantly upregulated in the 1 mg/ml LYG group (*P* < 0.05). Next, we verified that SOD was upregulated due to the upregulation of the sod-3 gene. Therefore, we conducted another assay with CF1553 (muIs84 [(pAD76) sod-3p::GFP + rol-6 (su1006)]); during heat stress, GFP (sod-3p::GFP) will be expressed in the head and tail and around the vulva. As shown in Fig. 4b–d, the fluorescence intensity that resulted from SOD-3::GFP expression in LYG-treated CF1553 worms was stronger than that in the control-treated worms (*P* < 0.01).

#### Effects of LYG treatment on the expression of aging-related genes

**Fig. 5:**
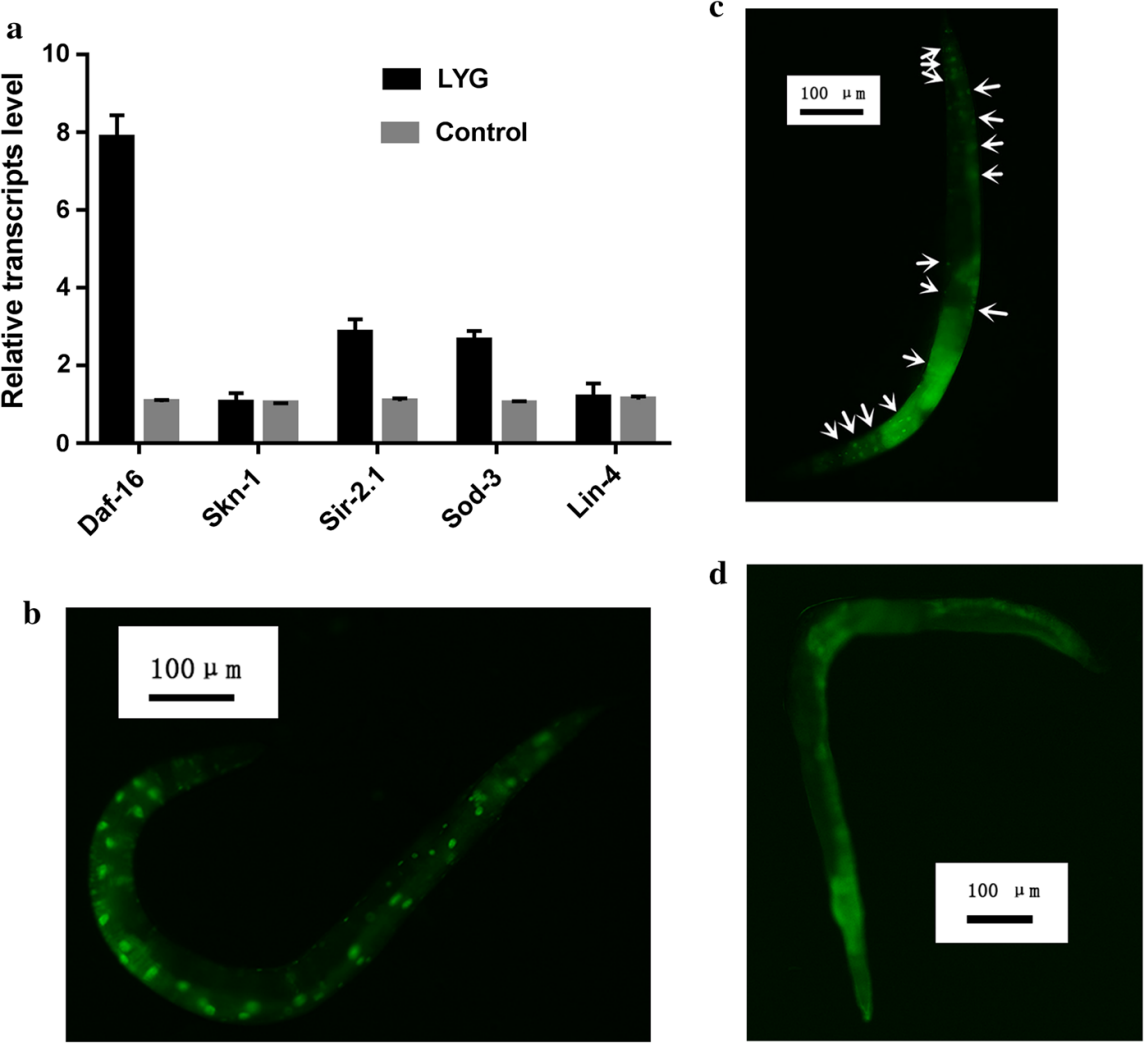
**a** The quantified relative expression of mRNA. **b** Effects of heat shock on the nuclear localization of daf-16. **c** Effects of LYG treatment on the nuclear localization of daf-16. **d** The control group

LYG treatment significantly increased the expression levels of the daf-16, sod-3 and sir-2.1 genes (*P *< 0.05, shown in Fig. 5a). To verify that the expression of the daf-16 gene was activated, we conducted another assay with the TJ356 strain (zIs356[daf-16p::daf-16a/b::GFP + rol-6]), which shows nuclear localization of daf-16 during heat stress, as indicated by GFP (daf-16::GFP) expression. To induce the nuclear localization of daf-16, worms were incubated at 36 °C for 2 h to induce heat shock. The worms were subjected to GFP expression analysis on the fourth day of adulthood. As shown in Fig. 5b–d, after the worms experienced heat shock, they showed the nuclear localization of daf-16. Similarly, the worms treated with LYG also showed the nuclear localization of daf-16, but the control worms did not show a change in daf-16 localization.

## Discussion

Restoratives in traditional Chinese drugs, such as *Ginseng* and *Radix Rehmanniae Preparata,* can attenuate age-related declines, as measured by several physiological and functional indices (Lee et al. 2007; Ko et al. 2010; Wan et al. 2014). Using the short-lived nematode *C. elegans*, we established a genetic system to examine the effects and the mechanism of LYG on longevity and aging. This work shows that treatment with LYG caused the extension of the mean adult lifespan as well as improved physiological function and stress tolerance. Finally, we showed that the beneficial effects of LYG treatment appear to require the activity of DAF-16/FOXO signaling, which also regulate stress resistance.

In modern times, an increasing number of age-related diseases, such as chronic degenerative diseases and other aging-related dysfunctions, have threatened human health (Gladyshev and Gladyshev 2016; Krut’ko et al. 2018). Contrary to mainstream modern medicine, TCM aims to interfere with the aging process as early as possible, thus preventing and delaying the occurrence and development of age-related diseases; this approach has begun to attract increasing attention from researchers (Liang and Yin 2010; Chen 2015). Moreover, many recent studies have focused on developing new products using herbal plants or compounds derived from these plants, which are considered to have low toxicity and to be free of adverse effects. For a long time, mixtures of medicinal plants have been used as traditional prescriptions in Eastern countries because the compounds in these mixtures have the potential to complement and enhance the effects of one another. According to TCM theory, LYG has reinforces Qi and nourishes Yin, as well as detoxifies the kidney and spleen, which are the main treatment strategies for aging (Hu 1986; Shen et al. 2002). Previous studies have shown that *Ginseng* and *Radix Rehmanniae Preparata* have antiaging effects; the active ingredients ginsenoside Rg1 (*from Ginseng*) and catalpol (from *Radix Rehmanniae Preparata*) can also exert antiaging effects (Chen et al. 2008; Zhang et al. 2008; Li et al. 2016). LYG is composed of *Ginseng* and *Radix Rehmanniae Preparata* and has been clinically proven to be effective, but further research is needed on the nature of LYG due to the complexity of its composition.

In this study, we explored the antiaging activity of LYG using a *C. elegans* model. We found that LYG treatment significantly prolonged the lifespan of wild-type worms under normal and stress conditions. This result indicates that LYG probably has good antiaging effects and preventative effects on age-related diseases. In addition, LYG treatment showed higher life-prolonging effects than its active ingredients ginsenoside Rg1 or catalpol (shown in Table 2), suggesting that the synergy of *Ginseng* and *Radix Rehmanniae Preparata* can lead to a stronger antiaging effect. Currently, the goal of antiaging medicine has shifted from simply extending lifespan to increasing health span. In this study, we showed that the age-related decline in functions, such as pharyngeal pumping, body movement, reproductive capacity and the reduction of lipofuscin accumulation, were effectively delayed in LYG-treated worms compared to control-treated worms, indicating that LYG could enhance the health span of worms.

To understand the underlying mechanisms by which LYG exerts antiaging effects, *C. elegans* was employed as an in vivo model, and SOD activity and the expression levels of aging-related genes were evaluated. Previous studies have suggested that the accumulation of oxidative stress caused oxygen free radicals and that this was a major factor in aging; thus, enhancing oxidative stress resistance could delay aging (Finkel and Holbrook 2000; Bokov et al. 2004). Our research indicated that LYG-induced the elevation of antioxidant enzyme activity, such as SOD activity, resulting in the elimination of oxygen free radicals, which might have antiaging effects. Previous studies have revealed that gene expression can change during aging in *C. elegans* (Murphy et al. 2003). LYG treatment might improve survival by activating the genes involved in the stress response, which affects lifespan in *C. elegans*. As shown in Fig. 4b–d, the fluorescence intensity of SOD-3::GFP expression in LYG-treated CF1553 worms was stronger than that in control worms, which suggests that LYG treatment increases the activity of SOD, probably via the activation of the sod-3 gene. Interestingly, a previous study suggested a possible causal association between antioxidant activities and longevity and a possible mechanism to account for the hormetic effects (Kitani et al. 2002; Calabrese et al. 2015). The daf-16 gene belongs to the “vitagenes family”, which is a group of genes strictly involved in preserving cellular homeostasis during stressful conditions (Calabrese et al. 2007); the antiaging effect of LYG could occur via a mechanism that involves the hormetic stimulation of a vitagene pathway. LYG may induce an adaptive stress response and activate adaptive cellular stress response pathways, such as Forkhead box protein (FOXO) transcription factors (daf-16 in *Caenorhabditis elegans*), which induce the expression of FOXO target genes (such as sod-3) that encode antioxidant enzymes (SOD) (Mattson 2008; Calabrese et al. 2011).

Furthermore, using qRT-PCR, we confirmed that the expression of the antiaging genes daf-16, sir-2.1 and sod-3 were upregulated in LYG-treated worms. LYG treatment might improve survival by activating the genes involved in stress responses that affect lifespan in *C. elegans*. The two transcription factors daf-16 and sod-3 can promote the expression of antioxidant or detoxification enzymes in *C. elegans*. The DAF-16/FOXO transcription factors promoted the expression of genes that confer extended longevity and enhanced stress resistance (Mukhopadhyay et al. 2006; Chavez et al. 2007). Sod-3, which is the downstream target of daf-16, promotes the expression of detoxification enzymes, such as SOD, in response to oxidative stress (Honda et al. 2010; Zhao et al. 2017). LYG treatment significantly increased the expression levels of the daf-16 and sod3 genes (Fig. 5a), further verifying that LYG regulated sod-3 downstream of daf-16 to extend lifespan and increase stress resistance. LYG treatment also significantly upregulated the expression level of sir 2.1 (shown in Fig. 5a), suggesting that LYG treatment can increase *C. elegans* lifespan through sir-2.1, which interacts with 14-3-3 proteins to activate daf-16 and extend lifespan (Berdichevsky et al. 2006). Sir-2.1 is another evolutionarily conserved regulator of longevity that increases the lifespan of *C. elegans* through either the downregulation of the insulin/insulin growth-factor signaling (IIS) pathway or the direct activation of daf-16 in parallel to IIS signaling. Furthermore, we conducted a nuclear localization assay with the TJ356 strain to further verify whether the daf-16 gene was activated. As shown in Fig. 5b–d, after experiencing heat shock, the worms showed the nuclear localization of daf-16. Similarly, the worms treated with LYG also showed the nuclear localization of daf-16, suggesting that LYG treatment exerted a life-prolonging and antiaging effect in *C. elegans* by activating the gene daf-16.

## Conclusions

In conclusion, this study demonstrates that LYG, a TCM formulation, increases stress resistance and promotes longevity in *C. elegans* via DAF-16/FOXO regulation.

## Data Availability

All the data are presented in the manuscript, and the authors declare that the submitted manuscript does not contain previously published data.
